# Study protocol for the development of a European measure of best practice for people with long term mental health problems in institutional care (DEMoBinc)

**DOI:** 10.1186/1471-244X-9-36

**Published:** 2009-06-13

**Authors:** Helen Killaspy, Michael King, Christine Wright, Sarah White, Paul McCrone, Thomas Kallert, Jorge Cervilla, Jiri Raboch, Georgi Onchev, Roberto Mezzina, Durk Wiersma, Andrzej Kiejna, Dimitris Ploumpidis, José Miguel Caldas de Almeida

**Affiliations:** 1 Department of Mental Health Sciences, University College London, 2nd Floor, Royal Free Hospital, London, NW3 2PF, UK; 2 Department of Mental Health, St George's University London, Cranmer Terrace, London, SW17 0RE, UK; 3 King's College London, Health Service and Population Research Department, Institute of Psychiatry, De Crespigny Park, London, SE5; 4 Department of Psychiatry and Psychotherapy, University Hospital, Technische Universitaet Dresden, Fetscherstrasse 74, 01307 Dresden, Germany; 5 Department of Psychiatry, University of Granada, Cuesta del Hospicio s/n, Granada, 18071, Spain; 6 Psychiatric Department of the 1st Faculty of Medicine, Charles University, Ovocný trh 5, Praha 1, 116 36, Czech Republic; 7 Department of Psychiatry, Medical University Sofia, St. Georgi Sofiisky str. 1, Sofia 1431, Bulgaria; 8 Dipartimento di Salute Mentale, Via Sai, 1-3, Trieste, 34127, Italy; 9 University Medical Centre, Hanzeplein 1, Groningen, 9700 RB, Netherlands; 10 Department of Psychiatry, Wroclaw Medical University, Pasteura 1, Wroclaw, 50-367, Poland; 11 University Mental Health Research Institute, Soranou Tou Efessiou 2, Athens 11527, Greece; 12 Caldas de Almeida, Faculdade de Ciências Médicas, Universidade Nova de Lisboa, Campo dos Mártires da Pátria 130, 1169-056 Lisbon, Portugal

## Abstract

**Background:**

This study aims to build a measure for assessing and reviewing the living conditions, care and human rights of people with longer term mental health problems in psychiatric and social care institutions. Protection of their human rights is imperative since impaired mental capacity secondary to mental illness can make them vulnerable to abuse and exploitation from others. They also constitute a major resource pressure for mental health services, social services, informal carers and society as a whole.

**Methods/Design:**

This study uses an iterative methodology to develop a toolkit to assess internationally agreed domains of care that are considered most important for recovery. These domains are identified by collating results from: i) a systematic review of the literature on institutional care for this service user group; ii) a review of the relevant care standards in each participating country; iii) Delphi exercises in partner countries with mental health professionals, service users, carers and advocates. Common domains and cross-cutting themes are agreed by the principal researchers and an international expert panel. Items are developed to assess these domains and incorporated into the toolkit which is designed to be administered through a face to face interview with the institution's manager. The toolkit is refined in response to inter-rater reliability testing, feedback from interviewers and interviewees regarding its utility, and feedback from key stakeholders in each country about its ability to deliver information that can be used within each country's established systems for quality assessment and review. Cross-validation of the toolkit ratings against service users' quality of life, autonomy and markers of recovery tests whether it can deliver a proxy-measure of the service users' experiences of care and the institution's promotion of their human rights and recovery. The ability of the toolkit to assess the "value for money" delivered by institutions is investigated by comparing toolkit ratings and service costs.

**Discussion:**

The study will deliver the first international tool for the assessment of the quality of institutional care for people with longer term mental health problems that is accurate, reliable, informative, useful and easy to use.

## Background

The study began in March 2007 and is funded for three years by the European Commission. It involves ten countries and aims to develop a toolkit for assessing and reviewing the living conditions, care and human rights of people with mental health problems who require longer term care in a psychiatric or social care institution. No such international, standardised assessment tools currently exist. The majority of people living in these kinds of institution have a diagnosis of schizophrenia or schizoaffective disorder [1], major mental illnesses which have a chronic or fluctuating prognosis in up to 60% of cases [2]. By definition, these individuals are also likely to have additional characteristics which have complicated their recovery such that they require ongoing institutional care. These may include: treatment resistance (persistence of psychotic symptoms despite appropriate medication) which occurs in up to 30% [3]; cognitive impairment, specifically in the areas of executive function, verbal memory and pervasive negative symptoms such as apathy, amotivation and blunted affect [4-7]; pre-morbid learning disability and poor function [8]; drug and/or alcohol misuse and challenging behaviours [4,9]. Despite their high levels of need and, possibly because of the complexity of their problems, there is very little evidence for effective interventions available to guide practitioners. As well as the significant clinical challenges they pose for professionals, these individuals constitute a major resource pressure for mental health services, social services, informal carers and society as a whole since they require high levels of support and are usually unable to work.

Many social care institutions that provide for this group are within the independent sector. In England, there has recently been considerable concern about the variability of care provided and social dislocation for the individuals placed in these institutions in terms of distance from their family home and dislocation from local communities, particularly for those from black and ethnic minorities [10-14]. This heterogeneity of service provision and quality is likely to be greater across countries.

Across Europe, countries are at different stages of deinstitutionalisation [15]. As such it is important that the toolkit covers themes that are both relevant and common to mental health services in different countries which are at different stages of development. Attention to the human rights of service users in these institutions is imperative since their mental health problems may impact on their capacity to make informed choices for themselves and to participate actively in their care. They are at risk of exploitation and abuse from others, including those who care for them. In addition, under resourcing of services can lead to practices which infringe civil liberties and deny basic human rights such as the right to privacy, choice and dignity. The European Commission's Green Paper "Improving the mental health of the population. Towards a strategy on mental health for the European Union" specifically highlights "the promotion of social inclusion of mentally ill or disabled people and protecting their fundamental rights and dignity". Quality of life and autonomy are key markers of whether clients' human rights are being promoted. Autonomy entails the freedom to choose from a range of options without persuasion or duress to influence that choice. However, the ability to choose can be affected by mental incapacity secondary to mental illness [16]. Quality of life is a global measure of an individual's well-being that can be affected by his or her human rights such as privacy, dignity, the absence of abuse and social inclusion [17]. Promotion of service users' autonomy should be a priority for these institutions since the negative effects of "institutionalisation" are well known [18].

### The Recovery framework

We chose to use the concept of "Recovery" as an overarching framework in the development of the toolkit since it incorporates these issues and offers a model for services that is relevant across different countries. The Recovery approach [19] specifically advocates non-coercive relationships between professionals and service users that include negotiation of treatment plans and facilitation of patient autonomy. The "Recovery movement" originated in the 1990s in America from service users' narratives challenging the pessimistic prognoses of mental health professionals. It focuses on the individual's adaptation to the mental illness and optimism for the future with the acknowledgement that pre-morbid functioning may not be fully regained [20]. It is corroborated by recent long term outcome studies which have shown encouraging results for the majority of people diagnosed with schizophrenia [21], even those with high levels of needs [9]. Mental health services are increasingly encouraged to adopt a "Recovery style" in their practice [22,23]. Features of Recovery based practice include: collaborative working between staff and service users; therapeutic risk-taking such as negotiated "drug holidays"; and service user empowerment. Service user markers of Recovery have also been suggested [24] such as working, studying and participating in leisure activities in mainstream settings; good family relationships; living independently; having control of one's self-care, medication and money; having a social life; taking part in the local community; and satisfaction with life. Some of these are also markers of social inclusion.

## Methods and design

### Rationale for the method

This study uses an iterative methodology to develop a toolkit to assess internationally agreed domains of institutional care relevant to commissioners and providers of services and service users. The initial stages of the study involve the identification of these domains of care and their components in order to conceptualise an "ideal" institution. The toolkit is designed to assess these components and domains of care and be further refined in response to piloting and reliability testing. Its ability to feed useful results into established systems to improve care is also assessed as part of the study and a health economic analysis using toolkit domain ratings to assess the "value for money" provided by the institutions is an additional component. The toolkit is designed as a structured questionnaire administered by face to face interview with the manager of the institution being assessed. This has the advantage of allowing an independent assessment of aspects of the institution (such as the décor and homeliness) by the interviewer. A later stage of the study involves the assessment of service users' quality of life, autonomy, satisfaction with care and markers of Recovery. If we find good correlation between the toolkit domain ratings and service users' experiences of the institution being assessed then we can be confident that the toolkit can provide a proxy measure of the institutions' promotion of service users' human rights and Recovery. This may mean that service user interviews will not be necessary in assessing institutions with the toolkit, which would maximise its practical application given the specific challenges that can arise in interviewing this service user group (secondary to their mental health problems). Having said this, assessors of institutions may, of course, wish to include interviews with service users. Our study design therefore aims to deliver a product (the toolkit) that allows flexibility in this to suit the specific setting.

### Setting and inclusion criteria

Our study is being carried out in ten European countries (Bulgaria, Czech Republic, Germany, Greece, Italy, Netherlands, Poland, Spain, Portugal and UK) selected to provide a range of different stages of deinstitutionalisation and a broad geographical and cultural spread across the European Union. It focuses on the institutional care received by adults with longer term mental health problems, the majority of whom have diagnoses such as schizophrenia and schizoaffective disorder. Institutions that specifically provide care for people with learning disabilities, developmental disorders or organic brain conditions including dementia are excluded in order to avoid the risk of carrying out a study with aims too broad to be able to deliver a meaningful result. We define an institution for potential inclusion in the study as a hospital or community based unit providing longer term care (i.e. not an acute admission unit) for at least seven service users in a communal setting (i.e. not individual flats/bedsits) with staff on-site, usually 24 hours a day (or if not 24 hours, the maximum number of hours provided in these kinds of units in that country). Units serviced only by outreach or "floating" support staff who are not based at the unit are excluded.

### Ethical approval

Each country submitted the study protocol to the relevant ethics committee for approval. In Italy ethical approval is not required for studies that do not involve invasive procedures. In the Netherlands, the study was discussed with the secretary of the ethics committee and considered to not require further formal approval by the full committee. For the UK centres the study was approved by the City and East London Multi Region Ethics Committee. The ethics committees that approved the study in the other participating countries were as follows: Germany (Ethik-Kommission der Medizinischen Fakultät Carl Gustav Carus an der Technischen Universität Dresden); Czech Republic (Ethics committee of the General University Hospital, Prague); Poland (Commission of Bioethics, Wroclaw Medical University); Bulgaria (Ethics Committee of the Medical University Sofia); Portugal (Ethics Committee of the Faculdade de Ciencias Medicas, New University of Lisbon); Spain (Comité de Ética de la Universidad de Granada); and Greece (University Mental Health Research Institutes Medical Ethics and Code of Conduct Committee, Athens).

## Description of the method

This study is being carried out in five phases.

### Phase 1: The identification of "critical success factors" for recovery and internationally agreed domains for the toolkit

Phase 1 involves two simultaneous work streams to identify the components and domains of care for inclusion in the toolkit. These two approaches were adopted to ensure that both quantitative and qualitative evidence were included in identifying domains and that the experiences and opinions of experts in the field (by nature of their personal or clinical experience) were included.

1) A systematic review of the peer reviewed literature published since 1980 will be carried out at the project co-ordinating centre in the UK, to identify components of institutional care provided for people with longer term mental health problems such as schizophrenia, and to examine the evidence for their effectiveness and efficacy. This literature review includes the search term "Recovery" and is enhanced by a "within country" review of care standards for institutions for people with longer term mental health problems carried out in each centre, the results of which will be collated by the co-ordinating centre;

2) A three stage Delphi exercise with four stakeholder groups in each country will be co-ordinated by the second UK centre, to gain common views on the most important aspects of care leading to recovery. The four groups will comprise individuals with personal or working experience of hospital or community based institutions for people with longer term mental heath problems and are as follows: mental health professionals (psychiatrists, social workers, nurses, occupational therapists, psychologists); service users; informal carers and relatives; and advocates of service users and carers. Ten to twelve participants will be recruited in each group in each country to ensure around ten from each group complete all three rounds. All materials for the Delphi exercise will be translated for use in each participating centre and back-translated. Back translations will be checked at the co-ordinating centre for any inconsistencies.

The Delphi question was agreed by the Principal Investigators at the first Project Steering Committee meeting in March 2007: "In your view, what most helps recovery for people with longer term mental health problems in institutional care?" (the definition of an institution is provided for respondents). In the first round respondents are asked to list a maximum of ten such components or factors. In the second round participants are asked to rate each item on the overall list of components in terms of their importance in contributing to recovery on a scale of 1 (unimportant) to 5 (essential). In the third round group members are asked to adjust their ratings in light of the median group scores for each item. These responses are then analysed for consensus agreement within groups, within and across countries.

Components of care that are identified as most important to recovery from these two work streams will then be grouped into common domains agreed by the Principal Investigators at each site and an international panel with expertise in Recovery, rehabilitation, human rights legislation, mental health legislation, the rights of people with mental disabilities and care standard setting. The membership of this international expert panel is detailed in the acknowledgements for this paper.

The toolkit will include descriptive items about the institution and its service users and items to assess these agreed domains of care. The content of items and number of items per domain will reflect the nature and relative importance of each domain as evidenced from the literature review, its inclusion in care standards across countries, the Delphi exercise results and the face validity of each item. Where possible the style and format of questions will be restricted so that the "best" answer is not obvious to the interviewee and answers can be entered into an algorithm whose output summarises the nature of the unit/institution being assessed. This does not restrict scores being used for individual domains. The toolkit will be further refined in response to suggestions from the Principal Investigators in each country and the International Expert Panel. Further items will be added using the same principles as described above.

### Phase 2: piloting and reliability testing the toolkit

The toolkit will be translated for use in each participating centre and back-translated. Back translations will be checked by the project co-ordinating centre and any inconsistencies identified and corrected. In each country, the toolkit will be piloted by the researchers in two institutions that meet the inclusion criteria (ideally one hospital and one community based). It will be refined after piloting to improve its accuracy by reviewing the semantic clarity of each item, avoiding duplication of items and items which are ambiguous or are two questions in one. Where data for items are unavailable for collection in one or more countries, the item will be discussed and its retention or removal agreed by consensus between Principal Investigators at each centre. Any points for clarification in the administration of the toolkit will be discussed at a researcher training session facilitated by HK and CW prior to Phase 2 recruitment starting. The toolkit will then be subjected to an evaluation of its inter-rater reliability in 20 institutions in each country. Institutions will be identified using existing records and registers in each country and recruited to provide a range of institutions that vary in: the proportion of voluntary (non-detained) and involuntary (detained) service users; size; geographic location (urban/rural); funding (statutory/non-statutory); gender of service users. The manager of each of the 20 institutions in each country will be interviewed by the researcher using the toolkit. Data for inter-rater reliability testing of the toolkit will be gathered by either having two raters present at the interview, repeating the interview with a second rater or tape recording the interview and having a second rater rate the recording. The reliability testing data analysis identifies items with poor spread and those with poor reliability.

### Phase 3. Refinement of the toolkit

The toolkit will be refined by consensus agreement with the Principal Investigators and International Expert Panel secondary to a) the results of the reliability testing and b) feedback from the researchers and interviewees regarding its usability and usefulness. Usability means the amount of time it takes to complete, the ease of completion and access to relevant information required for this. This information will be collected from researchers and service managers. The usefulness of the toolkit will be assessed through semi-structured feedback sheets completed by interviewees about the processes by which the findings could be used to improve care. The potential use of the toolkit for macro level systems change (e.g. feeding into regional or national care standards) will be assessed by discussion with key players working at this level in each country such as national leads and organisations involved in care standard setting. This multifaceted approach to refinement of the toolkit will maximise the chance of developing a useable and useful product able to deliver meaningful results.

### Phase 4: Association between toolkit assessments and service users' quality of life, autonomy and markers of recovery

In this phase of the study we will explore key associations between the toolkit assessment of institutions and service user experiences in terms of recovery and basic human rights. To do this we will first assess each institution by interviewing the unit manager using the refined toolkit and then evaluate the circumstances of a sample of ten service users within each institution. Service users of each institution will be recruited for interview to ensure a gender balance reflective of each institution. The manager of each institution will be asked to provide an anonymised list of their service users identifying each by a personal identification number and noting their gender. The researcher will select potential participants and inform the manager of the personal identification numbers. If the service user is willing to meet the researcher and informed consent gained, a face-to-face interview will be carried out at which assessment will be made of their quality of life, autonomy and experiences of care using standardised measures. If the service user declines or cannot participate, another service user, ideally of the same gender will be identified from the list and recruited. A minimum of five and a maximum of 13 service users will be recruited from all units in order to achieve a similar total per country. At least 20 units in each country must be included in Phase 4 (which can be the same units as participated in Phase 2) from which a minimum of 150 and a maximum of 200 service users are recruited for interview.

Quality of Life will be assessed using the Manchester Short Assessment of Quality of Life [25] which has been standardised for use in European countries. Autonomy will be assessed using a slightly adapted version of a tool used for people with learning disabilities living in residential care (the Resident Choice Scale [26]). There are no measures developed specifically for the assessment of autonomy of people with long term mental health problems. However, the issues relevant to those in longer term mental health institutions relate to mental capacity and the degree to which the institution promotes freedom of choice and independence across all aspects of everyday living. These aspects are captured in the Resident Choice Scale which requires only minor adaptation for our purposes. We will interview service users about their achievement of the "markers of Recovery" previously identified by Liberman and Kopelowicz [24] for people in institutional settings: working, studying and participating in leisure activities in mainstream settings; good family relationships; having control of self-care, medication and money; having a social life; taking part in the local community; and satisfaction with life and we will corroborate answers with information in the case notes and with key staff. Satisfaction with life will be assessed by the first question of the Manchester Short Assessment of Quality of Life [25]. We will also gather data on the service users' experience of care using the "Your Treatment and Care" questionnaire [27] which has been extensively used in service user led audits of inpatient and community mental health care in the UK. Payments to service user participants will be made (10 Euros) to compensate them for their time and trouble in undergoing face-to-face interviews. In previous work, we have found that small payments of this kind greatly facilitate the work of researchers and preserve the dignity of participants as partners in research. Such an approach has received support from a review of payments to participants in research [28].

The service user questionnaire will be translated for use in each participating centre and back-translated. Back translations will be checked by the project co-ordinating centre and any inconsistencies identified and corrected. In each country, the questionnaire will be piloted by the researchers with at least one service user. Any points for clarification are discussed at a researcher training session facilitated by HK prior to the start of Phase 4.

### Phase 5. Value for money

A health economic analysis of the "value for money" provided in each institution for different domains of care will be carried out. Data for this Phase will be collected concurrently with Phase 4 data collection. The cost of care received by service users is estimated in a three-stage process. First, service contacts for each service user recruited in Phase 4 will be recorded for the previous month (or six months for inpatient care) using a specially adapted version of the Client Socio-demographic and Service Receipt Inventory [29]. The professional within the institution with whom the user has contact is recorded, the number of such contacts, the reason (mental health or physical health) for them and whether they were individual or group contacts. In addition, key contacts from outside of the institution will be recorded. Second, unit costs of institutions (i.e. the cost per professional time) are estimated using expenditure data held by each institution with account taken of capital, administrative and other overheads. Unit costs of external services will be obtained where available or adapted from UK unit costs using appropriate adjustments based on WHO data [30]. Third, these unit costs are combined with the service use data to generate service costs for each user. User and institution characteristics that influence costs are identified using multilevel modelling. Value for money will be assessed by examining associations between institution and external service costs and the toolkit domain ratings.

### Data management

Common SPSS databases for all Phases that involve data collection will be developed in the UK centre co-ordinating that Phase and distributed to all centres. A test entry of data will be conducted in each centre before final modifications to the database are made. Appropriate range and logic checks are circulated by the co-ordinating centre to be carried out as part of data cleaning. Double data entry is completed for 10% of the data in a separate database distributed by the co-ordinating centre. The study statistician (SW) carries out data validation on the two databases for each centre. She will set a maximum error rate. If the centre's error rate is above this level then double data entry will need to be completed for all data at that centre. Where double data entry is required, a second database is sent to that centre by the co-ordinating centre for that Phase. Data validation on the two databases for each centre that has had to complete double data entry is carried out by SW and a report sent to these centres indicating where differences/errors in data entry have been made. Errors are then corrected in one master database for that centre which is returned to SW.

### Data Analysis

Data analyses for Phases 1, 2 and 4 are carried out by the study statistician.

#### Analysis of Delphi exercise data

Responses to Round 1 of the Delphi exercises will be collated and fed back to all respondents for rating on a scale of 1–5. Overall ratings are then re-rated by participants. Items rated in the top two categories ("essential" and "very important") are collated within country and consensus ratings measured. "Strong consensus" is defined as 100% of participants being within one point of the median score, and "consensus" as 80%. Consensus across partners is also analysed.

#### Analysis of reliability of the toolkit

1) Analysis of the inter-rater reliability of the specific items in the toolkit will involve the Kappa coefficient for categorical data (weighted Kappa where the number of categories is above two) and the intraclass correlation coefficient (ICC) for normally distributed, continuous data. In assessing the inter-rater reliability of a new instrument it is crucial to recruit sufficient paired ratings. Paired ratings for 20 institutions in 10 countries (200 institutions in all) in the reliability analysis will result in a sample that will enable a 95% confidence interval for the estimate of ICC of ± 0.15 [31]. We shall drop items whose weighted Kappa or ICC is below 0.4. Remaining items will be subjected to a principal components factor analysis in order to obtain the smallest number that will give most information and increase the internal consistency of the factors (subscales) arising. The scree plot will be inspected to identify the point at which factors should no longer be included. Internal consistency of the core and country-specific scales in the reduced toolkit will be assessed using Cronbach's alpha. We shall also explore the correlation of each item with the total score (item excluded), the average correlation with other items and Cronbach's alpha with that item removed.

#### Analysis of associations between toolkit ratings and ratings of service user quality of life, autonomy, satisfaction with care, markers of recovery and costs

Institutions and participating users will be described. The toolkit domain ratings will be analysed for associations with clients' quality of life, autonomy and markers of recovery to investigate whether the toolkit can provide a proxy measurement of the institution's promotion of human rights and recovery. A multilevel analysis of the data on core toolkit scores and all users from all countries will then be undertaken with the aim of delineating the main, independent predictors in the toolkit of patients' perceived autonomy, dignity, satisfaction with care, as well as potential markers for recovery. Multilevel models will allow the analysis of service user level data (level 1 units) of perceived autonomy, dignity, satisfaction with care, and markers of recovery as the dependent variable, to investigate how institution level toolkit domain ratings (level 2 units) relate. Similar models will be used to analyse the cost data. Although the final analysis will be carried out using this method there is scarce literature informing how to choose the number of level 1 units when the number of level 2 units is fixed. Given the aim of the analysis is to explore how the core toolkit domain ratings and the service users' human rights relate to each other, it appears sufficient to assume for the purposes of a power analysis that we have 200 units (institutions). Using Dunlap and colleagues [32], it has been calculated that to be able to test for 20 predictors of a medium effect size (R^2 ^= 0.35) with 80% power at a 5% significance level, a minimum of 170 units need to be included. These 20 predictors will be the resultant core toolkit domain ratings plus other possible institution and country level variables for which the analysis needs to be adjusted. Having multiple measures at each of these 180 units strengthens the power of the analysis since the multiple measures that will be used to estimate service users' autonomy, dignity, satisfaction with care, and markers of recovery will have less bias than a single service user measure.

A multilevel analysis of the pooled data on core toolkit scores for all users from all countries will then be undertaken. Levels likely to be important here include country, institution and service user. Using multivariable regression methods within the multilevel model will enable us to delineate the main, independent predictors in the toolkit of service users' perceived autonomy, dignity, satisfaction with care and markers of recovery.

## Discussion

The main output from this study will be the development of a robust toolkit for the assessment and review of the quality of care delivered to people with longer term mental health problems in hospital or community based psychiatric institutions in Europe. This is the first attempt to establish an international tool of this type. The multifaceted approach to identifying the items to be included in the toolkit (a broad systematic literature review, a qualitative Delphi exercise with key stakeholders and a review of the care standards in each participating country) and the further refinement of the toolkit though an iterative process that takes into account the results from piloting, reliability testing and the views of experts in the field, will ensure that the final product is accurate, reliable, informative, useful and easy to use. The toolkit will assess aspects of care that are important across countries with very different resources and at different stages of deinstitutionalisation and that reflect the institutions' promotion of the human rights and recovery of its service users.

The study findings will be prepared for publication and for presentation at national and international conferences. Local, national and international workshops for key stakeholders will be organised to disseminate the practical implications of the toolkit. We shall also discuss the findings from the study with the World Health Organisation, the Departments of Health and care standard setting agencies in the participating countries including the practicalities of incorporating the toolkit into existing review systems for institutional care. The development of a computerised algorithm that will provide rapid toolkit scoring for comparison of institutions, as well as individualised reports for each institution based on the toolkit assessment is a further potential output from this study. Such a report could be used for the unit's own audit purposes, for local, regional or national assessments of some or all the domains of care included in the toolkit. This algorithm could be adjusted in different countries to reflect the degree to which different elements of care might be expected to be developed depending on the stage of deinstitutionalisation of the country. This computerised version of the toolkit could be completed by unit managers without the need for a face to face interview to maximise its accessibility and could complement peer review or other face to face inspections.

The development of the DEMoBinc toolkit will have obvious benefits for the individuals living in institutions providing longer term mental health care since it will provide an objective assessment of the institution's practices and systems which have a direct bearing on service users' care and treatment. The toolkit will be of direct relevance to managers and commissioners of these services since it will provide assessment of a comprehensive range of key aspects of care. It will allow the identification of problem areas that require improvement and it will provide a means to review the effect of changes in practice. It will also provide a means for the assessment of "value for money" in relation to the different domains of care. It will be able to provide information on any number of health and social care institutions and could be used for regional or national surveys of institutional care and practices. In this way it will contribute directly to the review and setting of national and, potentially international, care standards for one of the most socially excluded groups of people in Europe.

## Competing interests

The authors declare that they have no competing interests.

## Authors' contributions

The original study idea was conceived by HK, CW and MK. All authors contributed to the further design of the study. All authors have been involved in drafting and/or revising the manuscript and have given final approval for the submission for publication of the final version.

## Pre-publication history

The pre-publication history for this paper can be accessed here:


